# Trace Elements in Scalp Hair Samples from Patients with Relapsing-Remitting Multiple Sclerosis

**DOI:** 10.1371/journal.pone.0122142

**Published:** 2015-04-09

**Authors:** Elisa Tamburo, Daniela Varrica, Gaetano Dongarrà, Luigi Maria Edoardo Grimaldi

**Affiliations:** 1 Dipartimento Scienze della Terra e del Mare (DiSTeM), University of Palermo, Palermo, Sicily, Italy; 2 U.O. Neurologia, Fondazione Istituto San Raffaele “G. Giglio” di Cefalù, Cefalù, Sicily, Italy; Friedrich-Alexander University Erlangen, GERMANY

## Abstract

**Background:**

Epidemiological studies have suggested a possible role of trace elements (TE) in the etiology of several neurological diseases including Multiple Sclerosis (MS). Hair analysis provides an easy tool to quantify TE in human subjects, including patients with neurodegenerative diseases.

**Objective:**

To compare TE levels in scalp hair from patients with MS and healthy controls from the same geographic area (Sicily).

**Methods:**

ICP-MS was used to determine the concentrations of 21 elements (Ag, Al, As, Ba, Cd, Co, Cr, Cu, Fe, Li, Mn, Mo, Ni, Pb, Rb, Sb, Se, Sr, U, V and Zn) in scalp hair of 48 patients with relapsing–remitting Multiple Sclerosis compared with 51 healthy controls.

**Results:**

MS patients showed a significantly lower hair concentration of aluminum and rubidium (median values: Al = 3.76 μg/g vs. 4.49 μg/g and Rb = 0.007 μg/g vs. 0.01 μg/g;) and higher hair concentration of U (median values U: 0.014 μg/g vs. 0.007 μg/g) compared to healthy controls. The percentages of MS patients showing hair elemental concentrations greater than the 95th percentile of controls were 20% for Ni, 19% for Ba and U, and 15% for Ag, Mo and Se. Conversely, the percentages of MS patients showing hair elemental concentrations lower than the 5th percentile of healthy controls were 27% for Al, 25% for Rb, 22% for Ag, 19% for Fe, and 16% for Pb. No significant association was found between levels of each TE and age, disease duration or Expanded Disability Status Scale (EDSS) score. After stratification by gender, healthy subjects did not show any significant difference in trace element levels, while MS patients showed significant differences (p<0.01) for the concentrations of Ag, Cr, Fe, Ni and Sr. No significant differences were also found, at p<0.01, in relation to the use of cigarettes, consume of water, vegetables and place of living.

**Conclusion:**

The different distributions of TE in hair of MS patients compared to controls provides an additional indirect evidence of metabolic imbalance of chemical elements in the pathogenesis of this disease. The increase in U and decrease in Al and Rb levels in MS compared to controls require further assessments as well as the observed different distributions of other elements.

## Introduction

Multiple sclerosis (MS) is a chronic neurological disease characterized by an idiopathic inflammation of the central nervous system (CNS) with lymphocyte and macrophage infiltration, leading to demyelination, axonal injury and the appearance of a variety of neurological signs over time [1, 2]. Although several evidences point to a clear genetic predisposition to MS, the north-south gradient of prevalence and familial studies of genetic inheritance suggest that an important role in the determination of the disease is also played by still unidentified environmental factor(s) [3].

Trace elements (TE) are crucial for the development of the nervous system, myelination of the nerve fibres, and neuronal excitability [4]. The ability of metals (redox-active or redox-inactive metals and even non metals as selenium) to promote neuronal damage and neurological dysfunction has been linked to their role in catalyzing the formation of superoxide anion O_2_^.-^, singlet oxygen ^1^O_2_* and hydrogen peroxide H_2_O_2_ [known collectively as *reactive oxygen species* (ROS)], which, in turn, are important mediators of oxidative reactions of biological macromolecules involved in neurodegenerative processes [5–14].

Abnormalities in transition metals and other TE in biological matrixes’ levels have been reported in several neurological diseases, including Alzheimer disease (AD), Parkinson disease (PD), amyotrophic lateral sclerosis (ALS) and MS [15–23]. Recently, the higher prevalence and incidence of MS observed among populations living in the eastern flank of Mt. Etna was attributed to their exposure to volcanogenic ashes containing TE, suggesting their role as possible environmental co-factor in the pathogenesis of MS [24]. However, the authors did not actually measured levels of TE in human samples and their conclusions were based on the comparison between two epidemiological distributions (MS prevalence and geochemical studies of volcanic gas emission).

To determine whether abnormal levels of TE were associated with MS, we measured 21 trace elements in the scalp hair of a cohort of Sicilian patients affected by relapsing–remitting MS (RRMS) compared to geographically-matched healthy controls.

## Methods

### Subjects

We studied 48 ambulatory RRMS patients (16 women and 32 men, mean age 35±8 years, all of them treated with natalizumab 300mg I.V. every 4 weeks) and 51 healthy controls (HC, 11 women and 40 men, mean age 48 ± 16 years) from cities representing all Sicilian provinces (Agrigento: *Agrigento*, *Licata*, *Palma di Montechiaro*; Caltanissetta: *Caltanissetta*, *S*. *Caterina Villarosa*, *San Cataldo*, *Sommatino*; Catania: *Caltagirone*, *Catania*, *Misterbianco*; Enna: *Enna*, *Nicosia*, *Pergusa*, *Villarosa*; Messina: *Ficarra*, *Gioiosa Marea*, *Messina*, *Patti*; Palermo: *Cefalù*, *Finale di Pollina*, *Gangi*, *Geraci Siculo*, *Palermo*, *Petralia Soprana*, *Sciara*, *Termini Imerese*, *Villabate*; Ragusa: *Giarratana*, *Modica*, *Ragusa*, *Scicli*; Siracusa: *Palazzolo Acreide*, *Rosolini*, *Siracusa*; Trapani: *Castellammare del Golfo*, *Marsala*, *Mazara del Vallo*). All patients were living in urban or closely sub-urban areas; the living environments were located 500 m a.s.l. for 19/48 MS patients (40%) and 19/51 for HC (38%) and at a lower altitude for 29/48 MS patients (60%) and 32/51 for HC (62%). Hair samples were collected at the MS Clinic of the Fondazione Istituto San Raffaele “G. Giglio” of Cefalù, Italy, between September 2011 and December 2012. All subjects were interviewed to obtain detailed information on their family, dietary habits, lifestyle and personal medical history. Patients and controls read and accepted the written informed consent approved by the local Ethical Committee “Fondazione Istituto San Raffaele—G. Giglio”, Prot. N. 2012/25. Biological samples were obtained according to the local existing legislation on privacy protection (D.M. n.675, 13/12/96) and the Declaration of Helsinki. Personal data were recorded and analyzed in an anonymous format.

Twenty pairs of samples were from subjects related to patients by direct line (first degree) of consanguinity or spouses, both sharing the same style of life. The remaining control subjects were friends of patients or community volunteers living in the same residence areas. Donors were randomly selected during one year time span and considered not eligible according to the following exclusion criteria:

- non-Caucasian ethnicity;
- ascertained respiratory, thyroid, kidney or liver diseases;
- recent surgery or orthodontic treatments;
- colored hair or recent use of hairstyling products.

Eighty percent of the possible participants, whose records did not support the defined characteristics, were excluded from the research study. Although the limited number of samples cannot adequately represent the whole population of MS patients in Sicily, estimated in about 6000 cases out of 5 million of inhabitants[25], MS donors were uniformly distributed among the nine Sicilian provinces (Agrigento: 5; Caltanissetta: 5; Catania: 5; Enna: 5; Messina: 5; Palermo: 12; Ragusa: 5; Siracusa: 4; Trapani: 3).

All subjects were interviewed to obtain detailed information on their date of birth, gender, residence and other personal background information (see S1 File). Smokers accounted for 43% and 25% among MS and HC, respectively; most of donors had an occupational activity, eleven were students; 75% and 54% commonly consumed bottled waters and vegetables, respectively. Most of them also claimed to live in an area with low vehicular traffic (Table 1).

**Table 1:** Biographical and clinical data.

	MS patient	MS (male)	MS (female)	HC control	HC (male)	HC (female)
**N**	48	32	16	51	40	11
**Age**						
Mean (SD)	35 (8)	37 (8)	30 (6)	47 (16)	51 (15)	34 (12)
min-max	20–53	20–53	20–40	19–78	22–78	19–63
**EDSS**						
Mean (SD)	2.4 (1.5)					
Median	2.0					
Score—N. Patients (%)						
0	1 (2.1%)					
1.0–1.5	17 (35.4%)					
2.0–2.5	16 (33.3%)					
3.0–3.5	4 (8.3%)					
4.0–4.5	6 (12.5%)					
5.0–5.5	1 (2.1%)					
>6	3 (6.3%)					
**Smokers**	43%	13%	7%	25%	9%	2%
**Vegetable consumers**	56%	52%	63%	52%	55%	45%
**Kind of water**						
Bottled water	71%	76%	63%	79%	75%	91%
Municipal water	11%	19%	18%	11%	17%	0%
Both	18%	5%	19%	10%	8%	9%
**Place of living**						
Low traffic	32%	64%	75%	76%	72%	90%
High traffic	68%	36%	25%	24%	28%	10%

Notes: N—Number of examined samples; SD—standard deviation; EDSS: Expanded Disability Status Scale. MS: Multiple Sclerosis patients and HC: healthy controls.

For the purpose of the present study we cumulated the results of all subjects in two separate groups (MS and HC). Biographical and clinical data are listed in Table 1.

### Sample collection and preparation

Strands of hair (~ 300mg), about 1–1.5cm long, were cut from the nape of the neck, as close as possible to the occipital region of scalp, and immediately stored in plastic bags, appropriately numbered and sealed together with an information form containing personal data as place of residence, age, gender, hair color and employment work. In the laboratory, the samples were reduced using a sterile surgical scalpel into smaller fragments to facilitate the subsequent washing procedure recommended by the International Atomic Energy Agency (IAEA), which consists of the sequence acetone-water-water-water-acetone [26–29]. More precisely, the samples were immersed in 20ml of acetone or water and each time stirred in ultrasonic bath for 15 minutes. After washing, the samples were placed in beakers and dried at low temperature (40°C) for 24h and then weighed. Then, 3 ml of HNO_3_ (Suprapur, Merck) were added to about 150mg of washed hair sample and digested for 24 h. Digestion was then completed by adding 500 μL of H_2_O_2_ (Suprapur, Merck) for a further period of 24h. Finally, the obtained solutions were diluted by the addition of 18 MΩ cm demineralized water to reach a volume of 25 mL. Quantification of 21 elements (Al, As, Ag, Ba, Cd, Co, Cr, Cu, Fe, Li, Mn, Mo, Ni, Pb, Rb, Sb, Se, Sr, U, V and Zn) was performed by inductively coupled mass spectrometry (ICP-MS, Perkin-Elmer, Elan 6100 DRC-e) after the addition of Re–Sc–Y as internal standards, using the technique of the additions to minimize the matrix effect. Determinations of As, Cr, Fe, Se and V were performed in DRC (Dynamic Reaction Cell) with reaction gases CH_4_ to reduce interferences induced by polyatomic ions generated by the plasma components, the solvent and the sample matrix. All standard solutions were prepared with 18 MΩ cm demineralized water, the ICP Multi Element Standard Solutions XXI CertiPUR, and the Mo and Sb CertiPUR standards (Merck). The analytical precision was estimated in the range 5–10% by running triplicate analyses on several samples. The IDL (instrument detection limit) was determined on the basis of three standard deviations (SD) of five blank measurements [30]. The validity of the analytical procedure was controlled by running reference materials QMEQAS08H-02 (hair collected from a single donor unexposed, spiked with selected analytes) Institut National de la Santé Publique—Laboratoire de Toxicologie, Quebec (Canada), and two reference materials made in our laboratory. The metal recovery rates of certified elements in the reference material ranged between 82% and 116%, with an average value of 98% (Table 2).

**Table 2:** Comparison of measured, reported concentrations and metal recovery of certified elements in standard reference material QMEQAS08H-02.

	QMEQAS08H-02	in this study	Recovery%
Ag	1.27±0.27	1.3±0.1	100
Al	31.9 ± 7.8	26.0 ± 1.55	82
As	3.45 ± 0.51	3.61 ± 0.35	105
Ba	3.58 ± 0.58	4.10 ± 0.43	115
Cd	3.54 ± 0.48	3.34 ± 0.12	94
Co	4.45 ± 0.51	4.41 ± 0.32	99
Cr	0.67 ± 0.289	0.53 ± 0.15	79
Cu	77.4 ± 0.72	73.3 ± 4.65	95
Fe	n.c.	12.5 ± 2.67	
Li	n.c.	0.09 ± 0.02	
Mn	1.87 ± 0.29	1.83 ± 0.89	98
Mo	1.01 ± 0.13	0.98 ± 0.11	97
Ni	5.12 ± 0.67	4.76 ± 0.42	93
Pb	13.2 ± 1.7	12.3 ± 0.67	93
Rb	n.c.	0.05 ± 0.004	
Sb	0.973 ± 0.164	1.13 ± 0.03	116
Se	1.20 ± 0.24	1.14 ± 0.17	95
Sr	n.c.	2.05 ± 0.11	
U	0.242 ± 0.053	0.22 ± 0.02	91
V	1.10 ± 0.17	1.17 ± 0.14	106
Zn	413 ± 60	434 ± 23	105

Data expressed as μg g^-1^;

n.c.—not certified.

### Statistical analysis

Undetectable measurements were substituted with 1/3 of standard deviation (SD) of five blank measurements. Data treatment was performed by a statistical approach using XLSTAT (Win 2010, Soft 32) [31]. Shapiro-Wilk test, with a level of significance set at p<0.01, was used to verify the normality of data distribution. Data were also examined to detect outliers by Dixon’s non-parametric test [32–34], as the data came from skewed populations. A total of 7 outliers (4 for MS and 3 for HC) were deleted from the database as they might unduly influence general results.

Differences between MS and HC groups were tested using the Mann–Whitney test, at p<0.01. Spearman’s rank correlation analysis was performed to estimate the degree of association between the metal levels in each group of hair samples. A cluster analysis (Agglomerative Hierarchical Clustering, AHC) was performed using raw data and Spearman’s coefficients as similarity criterion. We also decided to calculate the number of samples of each element for each group (MS and HC) that exceeded the 95^th^ percentile of concentration calculated for the other group. We considered 15% be the minimum number of samples exceeding the 95^th^ percentile as significant for the purpose of the present paper.

## Results

Results are listed in Table 3. The analysis of the controls (HC) showed that the most abundant hair elements were Zn>>Fe>Cu, the levels of these elements being from one to five orders of magnitude greater than all the other investigated TE. The median concentrations of Al and Sr were 4.49 and 1.70 μg/g, respectively; Se, Pb, Mn, Cr and Ni median values were between 0.1 and 1.0 μg/g, whereas the remaining elements had concentration levels below 0.1 μg/g. Although female controls exhibited higher median values compared to males for Ag, Cd, Co, Cu, Fe, Li, Mo, Ni, Pb, Rb, Sr, U, V and Zn, hair elemental concentration in females greater than the 95^th^ percentile of males was observed only for Zn (30%), Ni (24%), Mo (20%), Ag (18%), Sr (17%) and Co (15%). Hair samples from MS patients showed similar orders of abundance and magnitude (Zn>>Fe>Cu). When we studied gender differences in the MS group, we observed higher median values in female’s hair compared to males for Ag, Ba, Cd, Co, Cr, Cu, Fe, Li, Mn, Mo, Ni, Pb, Sb, Se, Sr, U, V and Zn.

**Table 3:** Basic statistical parameters of trace element contents in scalp hair from Multiple Sclerosis patients (MS) and healthy controls (HC).

MS										HC								
	Valid N	Mean	SD	Median	Q_5_	Q_25_	Q_75_	Q_95_	CV%	Valid N	Mean	SD	Median	Q_5_	Q_25_	Q_75_	Q_95_	CV%
Ag	46	0.1	0.17	0.03	0.01	0.02	0.12	0.53	155	47	0.1	0.06	0.03	0.01	0.02	0.07	0.18	102
Al	48	4.1	1.69	3.76	1.6	2.8	5.40	7.2	41	49	5.7	2.99	4.49	3.0	3.6	6.1	12.8	53
As	48	0.04	0.04	0.03	0.002	0.011	0.05	0.11	101	49	0.03	0.03	0.04	0.001	0.004	0.05	0.07	80
Ba	48	1.4	2.01	0.003	0.003	0.003	2.46	5.55	147	48	0.6	1.16	0.003	0.003	0.003	0.34	4.02	209
Cd	46	0.01	0.01	0.01	0.0003	0.002	0.01	0.03	101	51	0.01	0.02	0.01	0.0003	0.004	0.01	0.05	117
Co	48	0.1	0.12	0.02	0.01	0.01	0.07	0.37	161	51	0.1	0.15	0.02	0.01	0.02	0.06	0.53	187
Cr	48	0.6	0.63	0.30	0.06	0.10	0.71	2.13	114	51	0.7	1.51	0.21	0.04	0.12	0.40	3.36	215
Cu	46	11.7	3.6	10.2	7.6	9.3	13.1	18.8	31	51	11.2	4.2	10.3	7.3	8.9	11.4	22.3	38
Fe	48	16.4	9.50	14.6	6.0	8.9	20.9	37.6	58	49	16.4	8.25	14.1	7.8	10.6	19.5	35.5	50
Li	48	0.04	0.02	0.03	0.02	0.03	0.04	0.09	53	51	0.04	0.02	0.03	0.02	0.03	0.05	0.09	52
Mn	46	0.3	0.18	0.21	0.10	0.16	0.41	0.68	62	49	0.3	0.20	0.27	0.12	0.23	0.36	0.78	62
Mo	48	0.1	0.03	0.07	0.05	0.06	0.09	0.12	34	48	0.1	0.02	0.06	0.04	0.05	0.08	0.10	30
Ni	46	0.3	0.33	0.21	0.001	0.12	0.43	1.16	100	49	0.2	0.15	0.18	0.02	0.09	0.30	0.49	74
Pb	45	0.4	0.34	0.34	0.17	0.67	0.67	1.21	75	47	0.6	0.58	0.37	0.13	0.18	0.62	1.90	100
Rb	48	0.01	0.01	0.007	0.003	0.01	0.01	0.02	70	51	0.01	0.01	0.01	0.01	0.01	0.01	0.03	59
Sb	48	0.03	0.03	0.02	0.01	0.02	0.03	0.08	80	51	0.04	0.03	0.03	0.01	0.02	0.05	0.10	71
Se	48	1.0	0.69	0.70	0.41	0.50	1.2	2.4	70	51	0.8	0.47	0.66	0.38	0.51	1.1	1.9	56
Sr	46	4.6	5.10	2.3	0.35	1.04	6.2	15.6	112	49	2.8	2.73	1.7	0.33	0.73	3.9	9.7	97
U	48	0.03	0.04	0.014	0.001	0.005	0.03	0.13	152	48	0.01	0.01	0.007	0.001	0.001	0.01	0.03	134
V	45	0.1	0.05	0.05	0.01	0.02	0.08	0.17	83	48	0.1	0.05	0.05	0.01	0.03	0.08	0.17	71
Zn	48	211	54	209	134	179	230	346	26	51	191	48	190	117	159	225	265	25

Concentration data expressed as μg g^-1^ (dry weight basis). Notes: MS: Multiple Sclerosis patients and HC: healthy controls.SD—standard deviation, Q_5_, Q_25_, Q_75_ and Q_95_ indicate the 5^th^, 25^th^, 75^th^ and 95^th^ percentiles, respectively. CV indicates the coefficient of variation, calculated as: CV(%) = 100 × SD/mean.

Interestingly, female/male differences were more relevant in MS patients compared to healthy subjects. From a statistical point of view, healthy subjects did not show any significant difference when female and male were compared, while MS patients showed significant differences for concentrations of Ag, Cr, Fe, Ni and Sr (p<0.01) (Table 4).

**Table 4:** Results of Mann-Whitney test for differences between MS patients, HC controls and confounding variables.

	****p-value <0.05****	****p-value <0.01****
***Trace elements***		
MS + HC	Al (p: 0.005); Rb (p: 0.001); U (p: 0.005)	Al (p: 0.005); Rb (p: 0.001); U (p: 0.005)
**Age**		
MS	**-**	**-**
HC	Co (p: 0.028); Li (p: 0.009); Sr (p: 0.013); U (p:0.022)	Li (p: 0.009)
***Gender***		
MS	Ag (p:0.006); Cd (p: 0.011); Co (p: 0.017); Cr (p: 0.005); Fe (p: 0.001); Mo (p: 0.011); Mn (p: 0.016); Ni (p: 0.002); Pb (p:0.041); Sr (p: 0.001).	Ag (p:0.006); Cr (p: 0.005); Fe (p: 0.001); Ni (p: 0.002); Sr (p: 0.001).
HC	**-**	**-**
***Smokers***		
MS	Pb (p: 0.02); U (p: 0.04)	**-**
HC	**-**	**-**
***Vegetable consumers***		
MS	**-**	**-**
HC	As (p: 0.034)	**-**
***Place of living***		
MS	Ag (p: 0.044); Rb (p: 0.006)	Rb (p: 0.006)
HC	Mn (p: 0.017); Rb (p: 0.006)	Rb (p: 0.006)
**Kind of water (bottled water, municipali water, both)**		
MS	**-**	**-**
HC	**-**	**-**
***Expanded Disability Status Scale (EDSS)***		
MS	Cu (p: 0.022); Zn (p: 0.043)	**-**

We then compared cumulative data from MS patients with their geographically matched HC (Table 3). MS patients showed significantly lower Al (3.76 vs. 4.45 μg/g; p<0.01) and Rb (0.007 vs. 0.01 μg/g; p<0.01) and significantly higher U (0.014 vs. 0.007 μg/g; p<0.01) median concentrations (Table 3). The percentages of MS patients showing hair elemental concentrations greater than the 95^th^ percentile of controls were 20% for Ni, 19% for Ba and U, and 15% for Ag, Mo and Se. Conversely, the percentages of MS patients showing hair elemental concentrations lower than the 5^th^ percentile of healthy controls were 27% for Al, 25% for Rb, 22% for Ag, 19% for Fe, and 16% for Pb. No significant association was found between levels of each TE and age, disease duration or Expanded Disability Status Scale (EDSS) score. We also evaluated if smoking, the consume of water, vegetables and the extent of vehicular traffic of the place of living could affect elemental hair concentrations. No significant differences were found at p<0.01 (Table 4).

**Fig 1:**
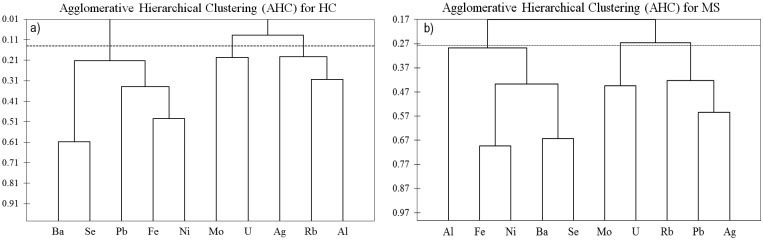
Agglomerative Hierarchical Clustering (AHC) performed using raw data. The degree of association is the Spearman’s coefficient.

On the base of the above findings and to evaluate possible associations among TE, two Cluster Analyses (AHC) were performed on each set of data (HC and MS) using the concentrations of Al, Ag, Ba, Fe, Mo, Ni, Pb, Rb, Se and U (Fig 1).

Three clusters were identified at a level of significance p<0.01 in HC (Fig 1a): in the first cluster (Ba-Se-Pb-Fe-Ni) we found a higher correlation between Se and Ba (r_Ba-Se_ = 0.63) and Fe-Ni (r_Fe-Ni_ = 0.53), while lower correlations were observed for Pb (r_Pb-Ni_ = 0.41, r_Ba-Fe_ = 0.40; r_Pb-Fe_ = 0.30); the second cluster (Ag-Rb-Al) showed milder associations (r_Rb-Al_ = 0.32; r_Ag-Rb_ = 0.23); the third cluster was made up of Mo-U which, however, were not significantly correlated (r_Mo-U_ = 0.21). Fig 1b shows that, at the same level of significance, the clustering in the scalp hair of MS subjects differed from HC. Namely, while the associations Fe-Ni, Ba-Se (r_Fe-Ni_ = 0.71, r_Se-Ba_ = 0.66;), Rb-Ag (r_Rb-Ag_ = 0.38) were still recorded, Mo-U exhibited a significant correlation (r_Mo-U_ = 0.44), the accumulation of Al was totally independent from the other TE and Pb was strongly correlated with Ag and Rb; r_Pb-Ag_ = 0.56, r_Pb-Rb_ = 0.50).

## Discussion

Multiple sclerosis is characterized by an autoimmune-mediated stripping and degradation of the myelin sheath that, at least initially, spare the nerve fibers within the CNS. Myelin sheaths are particularly susceptible to oxidative damage: since TE can increase free radicals production, it is conceivable that they may contribute to this demyelinating process Nicoletti et al. (2013)[24] evaluated the incidence of MS in two areas of the Mount Etna flanks with an allegedly different exposure to volcanic ashes carrying a high load of TE. Varrica et al. (2014)[35] evidenced that children living in the Mt. Etna area are naturally exposed to enhanced intakes of As, Mn, V and U, than those residing in other areas of Sicily, not influenced by volcanic activity, and indicated ingestion of water and local food as the most probable exposure pathways. We measured TE levels in human scalp hair samples of MS patients and geographically matched HC from the entire insular area of Sicily (25.711 square kilometers) and detected a relative greater abundance of Zn, Fe and Cu compared to the remaining TE in hair from both MS and HC individuals. The overexpression of these three key structural component of several proteins involved in cell protection against ROS and able to compete with other potentially obnoxious elements and metalloproteins for their binding sites [36, 37], is not surprising since it is a common feature of many highly reproducing tissues, including immune cells and hair [38–43].

Zn is an abundant metal in the human body being present in several organs, tissues, fluids and cells. It is an essential micronutrient being involved in a number of different reactions, among which the formation of zinc metalloenzymes, the metabolism of macronutrients, the processes of gene expression and replication of DNA molecules[44]. Zn also plays an important role as a constituent of metallothionine, a family of proteins possessing sulfur-based metal clusters, to which it is bound by the cysteine thiolate ligands. May be useful to recall that cysteine, along with histidine and methionine are aminoacids constituting the keratinic structure of hair.

Iron is present as haemoglobin in the erythrocytes of blood and it is carried by the plasma glycol-protein transferrin. It is essential for many metabolic processes, including the oxygen transport, DNA synthesis and electron transfer; iron is also a fundamental component of cytochromes acting in cellular respiration.

Copper is another essential and fundamental nutrient for living organisms being an important cofactor for many oxidative enzymes. It shows high affinity for aminoacids. Cu is necessary in the incorporation of iron in hemoglobin and it plays an essential role in the absorption of iron from the gastrointestinal tract and in its transfer from tissues to plasma[45].

It has been demonstrated that abnormality in the metabolism of Cu and Fe plays a crucial role in the pathogenesis of several neurodegenerative diseases; in particular, alterations in specific Cu and Fe containing metalloenzymes have been observed[46]. Copper can catalyze the production of hydroxyradicals and oxyradicals when it is available in the redox-active form Cu(II).

The ratio Zn/Cu is often indicated as a relevant ratio for the importance of the balance between these elements involved in the gene superoxide dismutase (SOD). Our results gave a range for Zn/Cu from 17 to 19, with no significant differences between HC and MS groups or between individuals differentiated by gender. This range, although obtained for a relatively small number of participants, such to preclude general conclusions, is clearly higher than the ratio 4–12 indicated as acceptable by Watts (2010) [47]. However, we have found similar high ratios in children hair from Sicily (Pace del Mela (ME): 14.5; towns located around Mt. Etna (CT): 14.9) [35, 43] as well as in literature (Zn/Cu: 18[48]; Zn/Cu: 19[19]; Zn/Cu: 15[49]). As suggested by Mattson et al. (2004) [46], abnormalities in the metabolism of Cu, especially when incorporated in metalloenzymes, may play a crucial role in the pathogenesis of several neurodegenerative diseases.

Interestingly, MS patients showed a significantly lower hair concentration of Al and Rb and higher hair concentration of U compared to HC. Are these observations just an epiphenomenon of other pathogenetic mechanisms or can they actually contribute to the pathogenesis of MS?

Aluminum is not considered essential for human life, although it is involved in the action of enzymes such as succinic dehydrogenase and δ-aminolevulinatedehydrase. A role for Al in neurodegenerative disease such as Alzheimer (AD) and Parkinson diseases has been hypothesized based on its ability to increase intracellular ROS in brain, its presence in senile plaques [50–56], its strong promotion of amyloid aggregation and accumulation [20, 57–59], a slight increase of its level in brain of AD patients, and several, although controversial, studies linking the amount of Al in drinking water to the incidence of AD[60, 61]. The use of the anti-oxidant and trivalent iron/aluminum chelator desferrioxamine has even been suggested as a treatment of AD [62, 63]. The additional ability, both in vitro and in vivo, of Al to promote inflammatory signaling via the pro-inflammatory transcription factor NF-kB may justify a role in the complex pathogenesis of MS[64, 65].

We actually found a significant decrease of Al in MS hair, thus apparently in contrast with the findings of Exley et al. (2006) [66] who detected increased excretion of Al in urines from MS patients, similar to what observed in people affected by aluminum intoxication or undergoing metal chelation therapy. Fulgenzi et al. (2012) [67] also reported that MS patients treated with the chelating agent calcium disodium ethylene diamine tetraacetic acid (EDTA) displayed elevated levels of aluminum in their urines. Although we did not assess urinary excretion of Al in our cohort, we speculate that the lower content of hair Al in our cohort of MS patients may reflect: 1) an accumulation of Al in target organs (brain?); 2) its increased elimination through urine leaving circulating Al in the bloodstream at levels insufficient to be uptaken by hair follicles.

Less is known about the role of Rb in human physiology and pathology. Chemically, Rb is a small atom behaving as K, their blood levels often fluctuating in parallel, almost always aggregated into more complex biological molecules. Rubidium is considered a non-essential metal and its biological role in the human body is still poorly understood. However, Rb has been found in blood, associated with red blood cells, at the same level of concentrations with Zn and Cu, which points to a possible essential role of this element in man [68]. Although nerve fibre refractory period was prolonged in depressive or schizophrenic patients treated with Rb, at present no effects are known on human immune function or neurodegeneration [69].

We found a lower concentration of Rb in hair of our MS cohort compared to HC. This depletion is similar to other reports in urine and blood of MS patients [16]. The lower content of Rb in MS can be the result of malabsorption and may reflect a metabolic disorder.

Uranium, finally, accumulates in human bones because of the crystallographic similarity of the uranyl ion (UO_2_^2+^) to that of calcium which allows uranium to replace calcium ions at the surfaces of bone mineral crystals [70, 71]. It is also deposited in kidney and liver [72]. The primary intake pathway of uranium is through inhalation, although ingestion and dermal contact can also contribute, and the main excretory pathway are the feces.

Our data show that the median U concentrations in hair of MS patients was significantly higher than in healthy individuals. Moreover, the percentage of MS patients showing U hair concentrations greater than the 95^th^ percentile of controls was 19%. Little is known about the concentration of uranium in the body and its involvement in the etiology of neurodegenerative diseases has yet to be demonstrated.

The correlation analysis also showed strong correlations Fe-Ni and Ba-Se. In the human body Fe and Ni are involved in enzymatic reactions by which the carbon monoxide dehydrogenase oxidizes CO to CO_2_ and the hydrogenase converts H^+^ ions to H_2_ molecular gas[73]. Differently, the association Se-Ba seems to have no definite explanation. 15% of MS patients showed hair Se concentrations greater than the 95^th^ percentile of controls. Selenium plays an essential biological role as part of the enzyme GSH-Px, forming one of the main antioxidant defense system. It is characterized by a narrow range between dietary deficiency and toxic levels and it is quickly excreted by urine and incorporated in blood and selenocysteine-containing proteins (cysteine is a main component of hair). Ba hair levels in MS exceeded the 95^th^ percentile of HC subjects in 19% of cases.

Purdey (2004) hypothesized that Ba ions could initiate the pathogenesis of MS breaking down the proteoglycan-FGF systems which sustains the oligodendrocytes and, as a consequence, hindering the synthesis or maintenance of the myelin sheath[74]. During a six-month longitudinal follow-up study carried out on trace elements in serum of MS patients, with first demyelinating episode, Visconti et al., 2005, recognized an increasing content of Ba in patients over the time and also respect to serum levels in healthy subjects living in the same geographic area[75].

Molybdenum exhibited a significant correlation with U in MS subjects; Mo is a redox active element, with oxidation states IV and VI, it is also an essential components of three classes of biological enzyme systems and an important cofactor for enzymes involved in catalyzing redox reactions on sulphur and nitrogen-containing compounds of DNA and RNA. It is also involved in the production of uric acid, and the oxidation and detoxification of various other compounds [76]. Accumulation of Mo may also contribute to the etiology of MS in some cases [77]. Visconti et al. (2005) [75] found higher Mo mean values in serum of patients affected by multiple sclerosis compared to controls, which seemed to be consistent with the relationship between Mo and MS reported by Zapadniuk (1992) [78].

## Conclusions

We have produced preliminary data on trace element concentrations in scalp hair samples from patients affected by RRMS and controls are provided. We found significant differences in Al, Rb and U hair concentrations, where U was significantly higher in MS patients while Al and Rb were higher in controls. Al concentrations were also particularly low in a significant number of hair samples from MS affected people. We could not ascertain whether the shortage of this element in hair is indicative of an accumulation in target organs. Additionally, female MS patients showed for a large number of trace elements a median hair content higher than males, with Ag, Cr, Fe, Ni and Sr being statistically different at p<0.01. These findings may indicate a possible relationship between MS pathology and deficiency of excess of distinct element species as well as alterations in their metabolism. The lack of strong correlation among TE concentrations and confounding factors as age, smoking, the consume of water, vegetables and the extent of vehicular traffic of the living place offers a good opportunity to differentiate HC and MS using element concentrations as discrimination variables.

Further studies using all possible matrices (including cerebrospinal fluid, neural tissues, blood, serum, and nails) and based on a larger number of patients and controls are required to confirm the uneven distribution of trace elements in multiple sclerosis and to assess its clinical relevance. In this context, special attention should be paid to the several molecules to which these patients are chronically exposed exposure for the treatment of their disease.

## Supporting Information

S1 File Format of the biographical data questionnaire. (DOC)

## References

[1] Ebers GC, Sadovnick AD. The role of genetic factors in multiple sclerosis susceptibility. J Neuroimmunol. 1994; 54:1–17. 7929798 10.1016/0165-5728(94)90225-9

[2] Sadovnick AD, Ebers GC, Dyment DA, Risch NJ, Canadian CSG. Evidence for the genetic basis of multiple sclerosis. Lancet 1996; 347:1728–1730. 8656905 10.1016/s0140-6736(96)90807-7

[3] Ebers GC. Chapter 6: The natural history of MS In: Paty DW, Ebers GC. Multiple sclerosis. 1998 Philadelphia: FA Davis and Co.

[4] Sadovnick AD, Ebers GC. Epidemiology in multiple sclerosis: A critical overview. Can J Neurol Sci. 1993; 20:17–29. 8467424 10.1017/s0317167100047351

[5] Stohs SJ, Bagchi D. Oxidative mechanisms in the toxicity of metal ions. Free Radical Bio Med. 1995; 18, 2, 321–336. 7744317 10.1016/0891-5849(94)00159-h

[6] Ercal N, Gurer-Orhan H, Aykin-Burns N. Toxic Metals and Oxidative Stress Part I: Mechanisms Involved in Metal-induced Oxidative Damage. Curr Top Med Chem. 2001 12; 1(6): 529–39. 11895129 10.2174/1568026013394831

[7] Valko M, Morris H, Cronin MTD. Metals, Toxicity and Oxidative Stress. Curr Med Chem. 2005; 12: 1161–1208. 15892631 10.2174/0929867053764635

[8] Clausen J, Jensen GE, Nielsen SA. Selenium in chronic neurologic diseases. Multiple sclerosis and Batten’s disease. Biol Trace Elem Res. 1998; 15:179–203. 10.1007/BF02990136 2484516

[9] Schiffer RB. Zinc and multiple sclerosis. Neurology 1994; 44: 1987–8. 7936271 10.1212/wnl.44.10.1987-a

[10] Le Vine SM. Iron deposits in multiple sclerosis and Alzheimer’s disease brains. Brain Res 1997; 760:298–303. 9237552 10.1016/s0006-8993(97)00470-8

[11] Fung YK, Meade AG, Rack EP, Blotcky AJ. Brain mercury in neurodegenerative disorders. J Clin Toxicol. 1997; 35: 49–54. 9022652 10.3109/15563659709001165

[12] Forte G, Bocca B, Senofonte O, Petrucci F, Brusa L, Stanzione P, et al Trace and major elements in whole blood, serum, cerebrospinal fluid and urine of patients with Parkinson’s disease. J Neural Transm. 2004; 111: 1031–40. 15254791 10.1007/s00702-004-0124-0

[13] Leonard SS, Harris GK, Shi XL. Metal-induced oxidative stress and signal transduction. Free Rad Biol Med. 2004; 37: 1921–42. 15544913 10.1016/j.freeradbiomed.2004.09.010

[14] Rivera-Mancía S, Pérez-Neri I, Ríos C, Tristán-López L, Rivera-Espinosa L, Montes S. The transition metals copper and iron in neurodegenerative diseases. Chem-Biol Interact. 2010; 186: 184–199. 10.1016/j.cbi.2010.04.010 20399203

[15] Ryan DE, Holzbecher J, Stuart DG. Trace Elements in Scalp-Hair of Persons with Multiple Sclerosis and of Normal Individuals. Clin Chem. 1978; 24: 11. 709834

[16] Schulten HR, Monkhouse PB, Achenbach C, Ziskoven R. Determination of rubidium in human serum. Experientia. 1983; 15; 39(7): 736–8. 6345189 10.1007/BF01990300

[17] Sayre LM, Perry G, Atwood CS, Smith MA. The role of metals in neurodegenerative diseases. Cell Mol Biol. 2000; 46: 731–41. 10875436

[18] Bomboi G, Marchione F, Sepe-Monti M, De Carolis A, Bianchi V, Medda E, et al Correlation between metal ions and clinical findings in subjects affected by Alzheimer’s disease. Ann Ist Super Sanità 2005; 41(2): 205–212. 16244394

[19] Forte G, Alimonti A, Violante N, Di Gregorio M, Senofonte O, Petrucci F, et al Calcium, copper, iron, magnesium, silicon and zinc content of hair in Parkinson’s disease. J Trace Elem Med Bio. 2005; 19: 195–201. 16325536 10.1016/j.jtemb.2005.08.003

[20] Exley C, House ER. Aluminium in the human brain. Monatsh Chem. 2010; 142: 357–363.

[21] Vinceti M, Solovyev N, Mandrioli J, Crespi CM, Bonvicini F, Arcolin E, et al Cerebrospinal fluid of newly diagnosed amyotrophic lateral sclerosis patients exhibits abnormal levels of selenium species including elevated selenite. Neurotox. 2013; 38: 25–32. 10.1016/j.neuro.2013.05.016 PMC3770807 23732511

[22] Nicoletti A, Patti F, Lo Fermo S, Messina S, Bruno E, Quattrocchi G, et al Increasing frequency of multiple sclerosis in Catania, Sicily: a 30-year survey. Mult Scler. 2011; 17:273–280. 10.1177/1352458510386995 21071466

[23] Valera P, Zavattari P, Albanese S, Cicchella D, Dinelli E, Lima A, et al A correlation study between multiple sclerosis and type 1 diabetes incidences and geochemical data in Europe. Environ Geoch Hlth. 2014; 36: 79–98. 10.1007/s10653-013-9520-4 23567975

[24] Nicoletti A, Bruno E, Nania M, Cicero E, Messina S, Chisari C, et al Multiple Sclerosis in the Mount Etna Region: Possible Role of Volcanogenic Trace Elements. PLOS ONE 2014; 8 Issue 12: 1–6. 10.1371/journal.pone.0074259 PMC3859652 24348986

[25] Giacoppo S, Galuppo M, Calabrò RS, D’Aleo G, Marra A, Sessa E, et al Heavy Metals and Neurodegenerative Diseases: An Observational Study. Biol Trace Elem Res. 2014; 161: 151–160. 10.1007/s12011-014-0094-5 25107328

[26] Sela H, Karpas Z, Zoriy M, Pickhardt C, Becker JS. Biomonitoring of hair samples by laser ablation inductively coupled plasma mass spectrometry (LA-ICP-MS). Int J Mass Spectr. 2007 261:199–207.

[27] Rodushkin I, Axelsson MD. Application of double focusing sector field ICP-MS for multielemental characterization of human hair and nails. Part II. A study of the inhabitants, of northern Sweden. Sci Total Environ. 2000; 262: 21–36. 11059839 10.1016/s0048-9697(00)00531-3

[28] Ryabukin YS. Activation analysis of hair as an indicator of contamination of man by environmental trace element pollutants. IAEA 1978; report IAEA/RL/50, Vienna.

[29] Subramanian KS. Determination of metals in biofluids and tissues: sample preparation methods for atomic spectroscopic techniques. Spectrochim Acta Part B. 1996; 51: 291–319.

[30] Birke M, Reimann C, Demetriades A, Rauch U, Lorenz H, Harazim B, et al Determination of major and trace elements in European bottled mineral water—analytical methods. J. Geochem. Explor. 2010; 107: 217–226.

[31] StatSoft, Inc. STATISTICA (data analysis software system), version 6. Available: http://www.statsoft.com.

[32] Dixon JW. Analysis of extreme values. Ann. Math. Stat. 1950; 21: 488–506.

[33] Barnett V, Lewis T. Outliers in Statistical data, 2nd. ed Wiley, New York 1984.

[34] Horn PS, Pesce AJ. Reference intervals: an update. Clin. Chim. Acta. 2003; 334: 5–23. 12867273 10.1016/s0009-8981(03)00133-5

[35] Varrica D, Tamburo E, Dongarrà G, Sposito F. Trace elements in scalp hair of children chronically exposed to volcanic activity (Mt. Etna, Italy). Sci Total Environ. 2014; 470–471: 117–126. 10.1016/j.scitotenv.2013.09.058 24126132

[36] Prasad AS, Bao B, Beck FW, Kucuk O, Sarkar FH. Antioxidant effect of zinc in humans. Free Radic Biol Med. 2004; 37: 1182–90. 15451058 10.1016/j.freeradbiomed.2004.07.007

[37] Goldhaber SB. Trace element risk assessment: essentiality vs. toxicity. Regul Toxicol Pharmacol. 2003 38: 232–42. 14550763 10.1016/s0273-2300(02)00020-x

[38] Creason JP, Hinners TA, Bumgarner JE, Pinkerton C. Trace elements in hair, as related to exposure in metropolitan New York. Clin. Chem. 1975; 21: 603 1116297

[39] Senofonte O, Violante N, Caroli S. Assessment of reference values for in human hair of urban schoolboys. J Trace Elements Med Biol. 2000; 14: 6–13. 10.1016/s0946-672x(00)80017-6 10836528

[40] Chojnacka K, Zielińska A, Górecka H, Dobrzański Z, Górecki H. Reference values for hair minerals of Polish students. Environ Toxicol Pharmacol. 2010; 29: 314–9. 10.1016/j.etap.2010.03.010 21787619

[41] Mikulewicz M, Chojnacka K, Gedrange T, Górecki H. Reference values of elements in human hair: A systematic review. Environ. Toxicol. Pharm.2013; 36:1077–1086 10.1016/j.etap.2013.09.012 24141206

[42] Dongarrà G, Lombardo M, Tamburo E, Varrica D, Cibella F, Cuttitta G. Concentration and reference interval of trace elements in human hair from students living in Palermo, Sicily (Italy). Environ Toxicol Pharmacol. 2011; 32: 27–34. 10.1016/j.etap.2011.03.003 21787726

[43] Dongarrà G, Varrica D, Tamburo E, D’Andrea D. Trace elements in scalp hair of children living in differing environmental contexts in Sicily (Italy). Environ Toxicol Pharmacol. 2012; 34: 160–9. 10.1016/j.etap.2012.03.005 22522426

[44] Falchuk KH. The Molecular Basis for the Role of Zinc in Developmental Biology, Mol. Cell Biochem. 1998; 188: 41–48. 9823009

[45] Murray RK, Granner DK, Mayes PA, Rodwell VW. Harper’s Biochemistry, 25th Edition, McGraw-Hill, Health Profession Division, USA 2000.

[46] Mattson MP. Pathways towards and away from Alzheimer’s disease. Nature 8 2004; 5; 430 (7000): 631–639. 15295589 10.1038/nature02621 PMC3091392

[47] Watts DL. HTMA Mineral Ratios. A brief discussion of their clinical importance. Trace Elem Newsletter 2010; 21: 1–3.

[48] Novak B. Contents and relationship of elements in human hair for a non-industrialised population in Poland. Sci Total Environ. 1998; 209: 59–68. 9496664 10.1016/s0048-9697(97)00298-2

[49] De Prisco PP, Volpe MG, Petitto F, Palladino C, Saturnino C, Capasso A, et al Level of essential and toxic metals in urban adolescents hair: Preliminary study. Biomed Res. 2010; 21: 131–140.

[50] Domingo JL. El aluminio como possible factor etiopatoge´nico en la enfermedad de Alzheimer. Rev Toxicol. 2000; 17: 3–11.

[51] Rondeau V. A review of epidemiologic studies on aluminium and silicon in relation to Alzheimer’s disease and associated disorders. Rev Environ Health. 2002; 17: 107–121. 12222737 10.1515/reveh.2002.17.2.107 PMC4764671

[52] Matsuzaki S, Manabe T, Katayama T, Nishikawa A, Yanagita T, Okuda H, et al Metals accelerate production of the aberrant splicing isoform of preselin-2. J Neurochem. 2004; 88: 1345–1351. 15009634 10.1111/j.1471-4159.2004.02290.x

[53] Gonzàlez-Munñoz MJ, Peña A, Meseguer I. Role of beer as a possible protective factor in preventing Alzheimer’s disease. Food Chem Toxicol. 2008; 46: 49–56. 17697731 10.1016/j.fct.2007.06.036

[54] Gonçalves PP, Silva VS. Does neurotransmission impairment accompany aluminium neurotoxicity? J Inorg Biochem. 2007; 101: 1291–1338. 17675244 10.1016/j.jinorgbio.2007.06.002

[55] Good PF, Olanow CW, Perl DP. Neuromelanin-containing neurons of the substantia nigra accumulate iron and aluminum in Parkinson’s Disease: a LAMMA Study. Brain Res. 1992b; 593: 343–346. 1450944 10.1016/0006-8993(92)91334-b

[56] Good PF, Perl DP, Bierer LM, Schmeidler J. Selective accumulation of aluminum and iron in the neurofibrillary tangles of Alzheimer’s disease: a laser microprobe (LAMMA) study. Ann Neurol. 1992a; 31: 286–292. 1637136 10.1002/ana.410310310

[57] Rodella LF, Ricci F, Borsani E, Stacchiotti A, Foglio E, Favero G, et al Aluminium exposure induces Alzheimer’s disease-like histopathological alterations in mouse brain. Histol Histopathol. 2008; 23: 433–439. 18228200 10.14670/HH-23.433

[58] Exley C. The aluminium-amyloid cascade hypothesis and Alzheimer's disease. Subcell Biochem. 2005; 38: 225–234. 15709481 10.1007/0-387-23226-5_11

[59] Walton JR, Wang MX. APP expression, distribution and accumulation are altered by aluminum in a rodent model for Alzheimer’s disease. J Inorg Biochem. 2009; 103: 1548–1554. 10.1016/j.jinorgbio.2009.07.027 19818510

[60] Flaten TP. Aluminium as a risk factor in Alzheimer’s disease. Brain Res Bull. 2001; 55: 187–196. 11470314 10.1016/s0361-9230(01)00459-2

[61] Frisardi V, Solfrizzi V, Capurso C, Kehoe PG, Imbimbo BP, Santamato A, et al Aluminum in the diet and Alzheimer’s disease: from current epidemiology to possible disease-modifying treatment. J. Alzheimers Dis. 2010; 20: 17–30. 10.3233/JAD-2009-1340 20378957

[62] Crapper McLachlan DR, Dalton AJ, Kruck TP, Bell MY, Smith WL, Kalow W, et al Intramuscular desferrioxamine in patients with Alzheimer’s disease. Lancet 1991; 337: 1304–1308. 1674295 10.1016/0140-6736(91)92978-b

[63] Percy ME, Kruck TP, Pogue AI, Lukiw WJ. Towards the prevention of potential aluminum toxic effects and an effective treatment for Alzheimer’s disease. J Inorg Biochem. 2011; 105: 1505–1512. 10.1016/j.jinorgbio.2011.08.001 22099160 PMC3714848

[64] Walton JR. Aluminum involvement in the progression of Alzheimer’s disease. J Alzheimers Dis 2013; 35: 7–43 Review Erratum in: J Alzheimers Dis 35: 875. 10.3233/JAD-121909 23380995

[65] Bondy SC. Prolonged exposure to low levels of aluminum leads to changes associated with brain aging and neurodegeneration. Toxicology 2013; 315: 1–7. 10.1016/j.tox.2013.10.008 24189189

[66] Exley C, Mamutse G, Korchazhkina O, Pye E, Strekopytov S, Polwart A, et al Elevated urinary excretion of aluminium and iron in multiple sclerosis. Mult Scler. 2005; 12: 533–40. 10.1177/1352458506071323 17086897

[67] Fulgenzi A, Zannella SG, Mariani MM, Vietti D, Ferero ME. A case of multiple sclerosis improvement following removal of heavy metal intoxication: lessons learnt from Matteo’s case. Biometals. 2012; 25: 569–576. 10.1007/s10534-012-9537-7 22438029

[68] Lombeck K, Kasperek LE, Feinendegen, Bremer HJ. Rubidium—A possible essential trace element. Biol. Trace Elem. Res. 1980; 2 193–198 10.1007/BF02785354 24271268

[69] Betts RP, Paschalis C, Jarratt JA, Jenner FA. Nerve fibre refractory period in patients treated with rubidium and lithium. J Neurol Neurosurg Psychiatry 1978; 41: 791–793. 690649 10.1136/jnnp.41.9.791 PMC493155

[70] Leggett RW. Basis for the ICRP’s age-specific biokinetic model for uranium. Health Phys. 1994; 67:589–610. 7960780 10.1097/00004032-199412000-00002

[71] Kurttio P, Komulainen H, Leino A, Salonen L, Auvinen A, Saha H. Bone as a Possible Target of Chemical Toxicity of Natural Uranium in Drinking Water. Environ Health Persp. 2005; 113: 1. 10.1289/ehp.7475 PMC1253712 15626650

[72] Parkhurst MA, Daxon EG, Lodde GM, Szrom F, Guilmette RA, Falo RE, et al Depleted Uranium Aerosol Doses and Risks: Summary of U.S. Assessments. Columbus, Ohio: Battelle Pres 2005.

[73] Watt RK, Ludden PW. Nickel-Binding Proteins, Cell. Mol. Life Sci. 1999; 56: 604–625. 11212309 10.1007/s000180050456 PMC11146914

[74] Purdey M. Chronic barium intoxication disrupts sulphated proteoglycan synthesis: a hypothesis for the origins of multiple sclerosis. Med Hypotheses. 2004; 62: 746–54. 15082100 10.1016/j.mehy.2003.12.034

[75] Visconti A, Cotichini R, Cannoni S, Bocca B, Forte G, Ghazaryan A, et al Concentration of elements in serum of patients affected by multiple sclerosis with first demyelinating episode: a six-month longitudinal follow-up study. Ann Ist Super Sanità 2005; 41: 217–222. 16244396

[76] Soetan KO, Olaiya CO, Oyewole OE. The importance of mineral elements for humans, domestic animals and plants: A review. Af J of Food Sci. 2010; 4: 200–222.

[77] Johnson. The possible role of gradual accumulation of copper, cadmium, lead and iron and gradual depletion of zinc, magnesium, selenium, vitamins B2, B6, D, and E and essential fatty acids in multiple sclerosis. Med Hypotheses. 2000; 55: 239–241. 10985916 10.1054/mehy.2000.1051

[78] Zapadniuk BV. The incidence of multiple sclerosis and the content of cobalt, boron, zinc, manganese and molybdenum in the arable soils of different climatic zones of Ukraine. Lik Sprava. 1992; 1: 111–3. 1364589

