# Supplementary material for: Trace Elements in Scalp Hair Samples from Patients with Relapsing-Remitting Multiple Sclerosis
Source: PLoS One. 2015 Apr 9;10(4):e0122142. doi: 10.1371/journal.pone.0122142 (PMC4391939; doi:10.1371/journal.pone.0122142)
Supplement: S1 File — (DOC) [file pone.0122142.s001.doc]

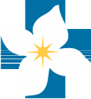


Fondazione Istituto **San Raffaele** - G. Giglio di Cefalù (Italy)

Dear Lady/Sir,

You are kindly invited to participate in this research study filling the following questionnaire. Your participation is voluntary, therefore you can choose whether or not you want to be enrolled in this study. All information collected on this form will be treated as strictly confidential and personal data will be recorded and analyzed in an anonymous format. These information are essential to study the relationships between trace elements and relapsing–remitting MS (RRMS).

**Name**:________________ **Surname** __________________ **Gender** male  female 

**Data of Birth: ____________________ Place of birth ____________________**

**Address (Street, City, Province) ________________________________________**

**Occupational activity :________________________**

**Hair colour:___________________________**

**Do you smoke cigarettes or cigars?** YES  (How many per day) ____ NO 

**What kind of water do you usually drink**? Bottled water  Municipal water  both 

**Do you eat vegetables?** YES  NO 

**You live in an area with:** high traffic  low traffic  no traffic 

**Have you orthodontic appliances?** **:** YES  NO 

**Are you suffering from other diseases?________________________________**

**Have you been exposed to any known occupational hazard?** YES  NO 

Thanks for your cooperation.
